# 874 Dilution Is Not Always the Solution: A Retrospective Study of Pulmonary Lavage in Inhalation Injury

**DOI:** 10.1093/jbcr/iraf019.405

**Published:** 2025-04-01

**Authors:** Ashleigh Bull, Colette Galet, Alexander Kurjatko

**Affiliations:** University of Iowa Burn Center; University of Iowa; University of Iowa Burn Center

## Abstract

**Introduction:**

Burned patients with inhalation injury commonly undergo bronchoscopy, at times with a thorough pulmonary lavage (PL). In animal studies, PL reduced the effects of primary injury of the smoke inhalation and the secondary inflammatory reaction. One study showed improved paO2 in humans. We characterized the outcomes of burned patients with inhalation injury who underwent PL at a single burn center.

**Methods:**

This is a retrospective cohort study. We queried our institution’s burn registry for all adult patients admitted between July 1, 2015 to June 30, 2023 who were on the ventilator and diagnosed with inhalation injury. Chemical inhalation, grade 0 inhalation injury, and diagnosis of inhalation injury without bronchoscopy were excluded. Demographics, burn size, burn location, hospital course information including length of stay (LOS) and ventilator days, use of PL, laboratory values, complications, and in-hospital mortality were collected. Categorical variables were compared using Chi-square and Fisher exact tests and continuous variables using the Mann-Whitney U test. Multivariate analyses were performed to identify variables, including PL, associated with outcomes. P < 0.05 was considered significant.

**Results:**

One hundred sixteen patients met inclusion criteria; 37 (31.9%) underwent PL. Most patients were male. Univariate analysis showed no significant differences in age, total body surface area burned (TBSA) or 2nd or 3rd degree TBSA, proportion of cases with complications, and in-hospital mortality between the no-PL and PL groups. The proportion of patients with chronic obstructive pulmonary disease was lower in the PL group than in the non-PL group (16.2% vs. 35.4%, p = 0.048). Patients in the PL group trended to be more likely to have an inhalation injury grade 2 or 3 (48.6% vs. 30.4%, p = 0.056). Patients in the PL group had more ventilator days (6 [2.5-15.5] vs. 2 [1-6], p < 0.001) and longer LOS (12 [4-37.5] vs. 5 [2-18], p = 0.003). Multivariate analysis, controlling for age, gender, inhalation injury grade, TBSA, and 2nd degree TBSA, showed that PL was associated with an increased time on a ventilator (OR = 1.84 [1.14-2.98], p = 0.013), increased hospital LOS (OR = 1.717 [1.080-2.730], p = 0.022), and increased risk of developing sepsis (OR = 7.216 [1.106-47.080], p = 0.039).

**Conclusions:**

In our cohort, PL was associated with longer ventilator days, longer LOS, and increased risk of sepsis. This was a single center retrospective study limiting its generalizability. Further multi-center studies are warranted to assess whether PL has benefits in burned patients with inhalation injury.

**Applicability of Research to Practice:**

Our results suggest that PL may not be beneficial in management of inhalation injury.

**Funding for the Study:**

N/A

